# Active living neighborhoods: is neighborhood walkability a key element for Belgian adolescents?

**DOI:** 10.1186/1471-2458-12-7

**Published:** 2012-01-04

**Authors:** Femke De Meester, Delfien Van Dyck, Ilse De Bourdeaudhuij, Benedicte Deforche, James F Sallis, Greet Cardon

**Affiliations:** 1 Department of Movement and Sport Sciences, Faculty of Medicine and Health Sciences, Ghent University, Ghent, Belgium; 2 Department of Human Biometry and Biomechanics, Faculty of Physical Education and Physiotherapy, Vrije Universiteit Brussel, Brussel, Belgium; 3 Department of Family and Preventive Medicine, University of California, San Diego, USA

## Abstract

**Background:**

In adult research, neighborhood walkability has been acknowledged as an important construct among the built environmental correlates of physical activity. Research into this association has only recently been extended to adolescents and the current empirical evidence is not consistent. This study investigated whether neighborhood walkability and neighborhood socioeconomic status (SES) are associated with physical activity among Belgian adolescents and whether the association between neighborhood walkability and physical activity is moderated by neighborhood SES and gender.

**Methods:**

In Ghent (Belgium), 32 neighborhoods were selected based on GIS-based walkability and SES derived from census data. In total, 637 adolescents (aged 13-15 year, 49.6% male) participated in the study. Physical activity was assessed using accelerometers and the Flemish Physical Activity Questionnaire. To analyze the associations between neighborhood walkability, neighborhood SES and individual physical activity, multivariate multi-level regression analyses were conducted.

**Results:**

Only in low-SES neighborhoods, neighborhood walkability was positively associated with accelerometer-based moderate to vigorous physical activity and the average activity level expressed in counts/minute. For active transport to and from school, cycling for transport during leisure time and sport during leisure time no association with neighborhood walkability nor, with neighborhood SES was found. For walking for transport during leisure time a negative association with neighborhood SES was found. Gender did not moderate the associations of neighborhood walkability and SES with adolescent physical activity.

**Conclusions:**

Neighborhood walkability was related to accelerometer-based physical activity only among adolescent boys and girls living in low-SES neighborhoods. The relation of built environment to adolescent physical activity may depend on the context.

## Background

Increasing physical activity in youth is one of the key public health strategies to conquer the alarming rise of overweight, obesity and a cluster of risk factors associated with cardiovascular disease and type 2 diabetes [1,2]. To achieve substantial health benefits for school-aged youth, participation in physical activity of at least moderate to vigorous intensity for a minimum of 60 minutes per day is recommended [3,4]. A large proportion of school-aged youth does not achieve the public health recommendations [5-8]. In addition, adolescence is marked by a decline in time spent in physical activity which is more apparent in adolescent boys than in adolescent girls [9-11].

Ecological models provide a framework for understanding the multiple facets that influence physical activity. From the perspective of ecological models, an interwoven relationship between individual and psychosocial, sociocultural, policy and physical environmental factors influences behaviors [12]. Past research has identified demographic (e.g. age, gender, pubertal status), psychosocial (e.g. self-efficacy, perceived competence, parental and peer support), sociocultural (e.g. ethnicity) and policy (e.g. extracurricular physical activity) correlates of physical activity among adolescents [13-17]. More recently, the importance of the physical environment as an opportunity to shape physical activity has been established [18-21].

An important construct among the physical environmental correlates is neighborhood "walkability". Neighborhoods considered walkable are characterized by mixed land use, well-connected streets and high residential density [22,23]. These elements are synergistic and can be objectively determined using Geographic Information Systems (GIS) software [24]. The research into the relationship between neighborhood walkability and physical activity has only recently been extended to young people and the current empirical evidence is not consistent [25-28]. The review of Ding et al. [29] established that in only 20% of the studies that investigated the association between objectively determined neighborhood walkability and objectively determined physical activity among adolescents, a positive association was found. Ding et al. stated that when investigating the association between neighborhood environment and youth physical activity, conclusions based on objectively measured environmental attributes seem more credible because of the lower measurement error associated with objective measures. Furthermore, it was stated that self-reported physical activity that captures specific domains of activity allow for tests of association between conceptually matched environmental and physical activity variables.

In research on adults, neighborhood "walkability" has been supported as a key construct among the built environmental determinants. Four studies with a similar design investigated the relationship between objectively determined neighborhood walkability and physical activity in adults: the Neighborhood Quality of Life Study (NQLS) conducted in the US [30], the Physical activity in Localities and Community Environments (PLACE) study conducted in Australia [31], the Belgian Environmental Physical Activity Study (BEPAS) [32] and the Swedish Neighborhood and Physical activity (SNAP) study [33]. The four studies examined neighborhood socio-economic status (SES) as a possible moderator of the association between neighborhood walkability and physical activity. For each study, participants were recruited from "quadrants" of neighborhoods; low-SES/high-walkable, low-SES/low-walkable, high-SES/high-walkable and high-SES/low-walkable neighborhoods were defined to ensure diversity of environments. Living in neighborhoods characterized by higher walkability was found to be associated with more walking for transport [30-33], more cycling for transport [32], more walking for leisure [30,32,33] and more accelerometer-based moderate to vigorous physical activity [30,32,33].

The results of the four studies concerning the moderating effect of neighborhood SES on the association between neighborhood walkability and physical activity were not totally comparable. In NQLS, the association between neighborhood "walkability" and walking for transport was stronger in high-SES than in low-SES neighborhoods [30]. In PLACE, BEPAS and SNAP the benefits from neighborhood "walkability" were similar in high- and low-SES neighborhoods [31-33].

The results of studies in youth, investigating the direct association between neighborhood SES and physical activity, showed a positive association between neighborhood SES and physical activity [34-36]. However, to our knowledge, the study of Kerr et al. [37] was the only study investigating the interaction between neighborhood walkability and neighborhood SES in youth. The results of this US study revealed that physical activity behavior of 5-18 year olds reported by the parents was related to objectively determined neighborhood walkability in high-income neighborhoods and not in low-income neighborhoods.

Considering the discrepancies in needs and behaviors between adults and youth, the relationships found in adults may not be generalisable to youth. Youth are dependent on adult rules governing travel and destination choices and are not licensed to use motor vehicles under the age of 16. Consequently, they are more captive in their own neighborhood, and the influence of local neighborhood environmental attributes may be more pronounced in youth. Therefore, to design an active living neighborhood built environment suitable for both youth and adults, urban planners should know whether and how neighborhood walkability is related to physical activity among adolescent boys and girls.

The aims of the Belgian Environmental Physical Activity study in Youth (BEPAS-Y) were (A) to investigate the association between neighborhood walkability, neighborhood SES and physical activity in youth (B) to investigate whether the association between neighborhood walkability and physical activity is moderated by neighborhood SES and gender.

## Methods

BEPAS-Y was a cross-sectional study conducted in Ghent. Ghent, the capital of the Belgian province East-Flanders occupies over 156.18 sq km (60.3 sq miles) with 1,554.40 inhabitants per sq km (2009). The research protocol of BEPAS in adults [32], which builds on the protocols of NQLS [30] and PLACE [31], was used as template for BEPAS-Y. BEPAS-Y received approval from the Ethics Committee of Ghent University Hospital.

### Selection neighborhoods

**Figure 1:**
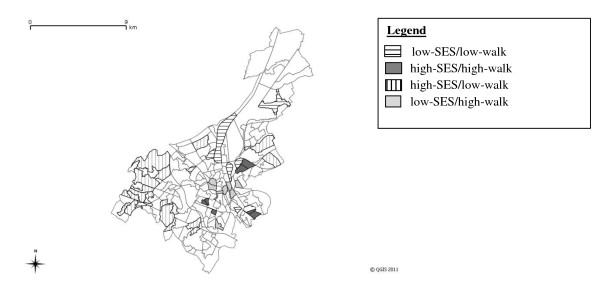
**Distribution of neighborhoods in Ghent, Belgium**. The distribution of the 32 selected neighborhoods in Ghent, Belgium: 8 high-walkable/low-SES, 8 high-walkable/high-SES, 8 low-walkable/low-SES, and 8 low-walkable/high-SES.

Ghent consists of 201 statistical sectors, the smallest administrative entities for which statistical data produced by the Belgian National Institute of Statistics (NIS) are available. Belgian census data derived from the NIS were used to define SES, and geographical information derived from the available GIS databases was used to define walkability. To obtain neighborhoods with a sufficient number of inhabitants as a recruitment pool (approximately 1,000) [30,32], adjacent statistical sectors characterized by comparable walkability (within the same quartile based on the walkability index) and SES (within the same decile based on the median annual household income) were used to define a neighborhood. Consequently, the geographical area of low-walkable neighborhoods was larger than of high-walkable neighborhoods (1.8 km^2 ^vs. 0.4 km^2^), and the average population density was lower (1535.6 inhabitants/km^2 ^vs. 6201.2 inhabitants/km^2^). The selection process involved two steps: 1) the statistical sectors were stratified on neighborhood walkability (GIS-based) and SES and 2) neighborhoods (one or more adjacent statistical sectors) meeting criteria for high/low walkability and high/low SES were identified. The selection resulted in 32 neighborhoods: 8 high-walkable/low-SES, 8 high-walkable/high-SES, 8 low-walkable/low-SES, and 8 low-walkable/high-SES (Figure 1). Each neighborhood comprised 1-5 contiguous statistical sectors.

#### Neighborhood walkability

For each statistical sector a walkability index was calculated using three objective GIS-based measures: residential density, intersection density, and land use mix, which have been consistently related to physical activity [38,39]. Geographical cadastral data (residential land use, street centerline data, zoning data) and census data provided by the Service for Environmental Planning in Ghent were integrated in a GIS database and used to determine the walkability components.

Net residential density represents the ratio of residential units to the land area devoted to residential use per statistical sector. Connectivity is the ratio between the number of true intersections (three or more legs) to the land area of each statistical sector. Land use mix is an indication of the degree to which a diversity of land use types were present in each statistical sector. Five land uses were considered: residential, retail (supermarkets, bakeries, butchers, banks, and clothing shops), office, institutional, and recreational (sport and non-sport). The corresponding values were normalized and z-scores were calculated. The three-component walkability-index was created by weighing the z-scores of the environmental features, using the following expression: walkability = (2*z-connectivity) + (z-residential density) + (z-land use mix). The formula used is an adapted version of the formula of Frank and colleagues [40]. Because no GIS data were available, "retail floor area ratio" was omitted from the formula. Based on their walkability index, the statistical sectors were ranked and quartiles were constructed. The highest quartile constituted the high-walkable sectors, the lowest quartile the low-walkable sectors.

#### Neighborhood SES

The socioeconomic environment of each statistical sector was defined in terms of the median annual household income, using data from the NIS (Belgium, 2008). Corresponding to their income data, the statistical sectors were ranked and divided into deciles. Outliers were avoided by excluding sectors with annual household income values less than €11,600 and greater than € 116,000. The second, third, and fourth deciles of the ranking contained the low-SES sectors; the seventh, eighth, and ninth deciles the high-SES sectors [32].

### Procedure

After neighborhood selection, the addresses from all, 13-15 year old adolescents (n = 1553) living in the selected neighborhoods were acquired by the Public Service of Ghent. Between October 2008 and May 2009 an informative letter about the study with an invitation to participate was posted to the potential participants. One week later, home visits took place. Participants were recruited simultaneously in the four groups of neighborhoods throughout the recruitment period to avoid seasonal bias.

In May 2009, 1,399 adolescents were invited, 1,078 adolescents were found at home and 59.1% (n = 637; 49.4% boys) consented to participate. Because at that moment, recruitment goals were achieved with approximately equal numbers of participants within the four groups of neighborhoods, it was decided not to contact the remaining 154 adolescents as they were mostly from high SES neighborhoods. Written consent was obtained from all participants and the adolescents' parents or legal guardian. During the home visit, a questionnaire was delivered and an interview was conducted. The protocol of the accelerometer and non-wear activity diary was explained and an appointment for a second home visit was made to collect the accelerometer, diary and questionnaire.

### Measures

#### Physical activity

##### Objectively assessed physical activity

Physical activity was objectively assessed using accelerometers, model GT1M (Actigraph MTI, Manufacturing Technology Inc., Pensacola, FL, USA) and model 7,164 (Computer Science Application, Inc., Shalimar, FL, USA). Two recent studies confirmed that the output of accelerometers model GT1M and model 7,164 was similar and that therefore the two models can be used in the same study [41,42]. The adolescents were asked to wear an accelerometer during waking hours, for 7 consecutive days including 2 weekend days. Secured by an elastic belt, the accelerometers were worn on the right hip, above the iliac crest.

Non-wear time activity diaries were provided to register activities for which the accelerometer was removed (aquatic activities or activities that prohibit an accelerometer). Adolescents recorded on a pre-printed form when the accelerometer was removed, when they put it back on, and the kind of the activities they were involved in [43,44].

Data-reduction software, MeterPlus 4.2. [45], was used to screen, clean and score the accelerometer data. In the data reduction process, time periods of at least one hour of consecutive zeros were removed, assuming the accelerometer was unworn [46,47]. Whenever applicable, these consecutive number of zeros were, after the accelerometer data scoring process, replaced by the corrected number of minutes moderate physical activity and vigorous physical activity registered in the diaries [43,44]. To score the accelerometer data, the thresholds of Puyau (moderate physical activity: 3,200-8,199 counts/min and vigorous physical activity: ≥ 8,200 counts/min; respectively corresponding to activities 3-6 MET and activities > 6 MET) [48] were used. For inclusion in the data analysis, the required total accumulated number of minutes registered time by the accelerometer and diaries was 600 minutes for weekdays and 480 minutes for weekend days. Furthermore, 3 valid weekdays and 1 valid weekend day of monitoring were needed to obtain reliable estimates [49-51]. The accelerometer data were used to provide an indication of adolescents physical activity by means of the average activity level expressed in counts/minute (CPM) and mean minutes of moderate to vigorous physical activity per day (MVPA). The average activity level gives an indication of the total level of physical activity and is not dependent of the chosen cut-points. The number of minutes of moderate to vigorous physical activity is an outcome that is relevant for the assessment of activity relative to meeting public health guidelines.

##### Self-reported physical activity

The Flemish Physical Activity Questionnaire (FPAQ) [52] (interview version) was used to determine the duration (hours and minutes per day) of specific physical activity behaviors undertaken in specific contexts: school related active transportation (walking and cycling to and from school), walking and cycling for transport during leisure time, and sport during leisure time. The FPAQ was found to be a reliable and reasonably valid questionnaire for the assessment of different dimensions of physical activity in 12-18 year old adolescents [52].

#### Demographic variables

Self-reported data included adolescents' gender, age, nationality and SES. Educational attainment and employment of the adolescent' parents were used as a proxy measure of adolescents' SES. The educational level of the adolescents' mother and father was determined based on four options: less than high school, completed high school, completed college or completed university. The employment status of mother and father (employed, unemployed) was coded into both parents employed, one of the parents employed or both parents unemployed.

### Statistical analyses

To provide information about characteristics of the sample, descriptive statistical analyses were conducted using SPSS 17.0. Tests for normal distribution revealed some skewed physical activity variables. To obtain distributions that more closely approximated symmetry, logarithmic transformations were conducted, and the transformed variables were used in the analyses. For ease of interpretation, summary data of untransformed physical activity variables are reported in minutes/day.

To analyze the associations between neighborhood walkability (dichotomous variable: low/high neighborhood walkability), neighborhood SES (dichotomous variable: low/high neighborhood SES) and physical activity, multivariate regression analyses were conducted using MLwin version 2.22. To examine if the association between neighborhood walkability and physical activity behavior was moderated by neighborhood SES, the cross-product term "neighborhood walkability × neighborhood SES" was entered in the regression model.

To examine if the association between neighborhood walkability, neighborhood SES and physical activity behavior was moderated by gender, the cross-product terms "neighborhood walkability × gender", "neighborhood SES × gender" and "neighborhood walkability × neighborhood SES × gender" were separately included in the regression model.

All analyses were controlled for three proxy measures of individual SES (parental employment and educational attainment of mother and father) [53]. Clustering of individuals in neighborhoods was taken into account by using multi-level modelling with adolescents at the first level and neighborhoods at the second level. Neighborhood-level attributes (neighborhood walkability and SES) were handled as level-2 variables, individual attributes (physical activity, parental employment and parental education) were handled as level-1 variables.

## Results

From the 637 adolescents who consented for the study, 615 (96.5%) returned a complete physical activity questionnaire, and 513 (80.5%) had complete accelerometer data. Table 1 represents descriptive statistics for demographic characteristics by type of neighborhood. Mean age of the total sample was 14.6 ± 0.9 years, and 50.4% was female. Most adolescents' parents were highly educated, as 60.8% attained college or university, and most parents were both employed (68.5%).

**Table 1:** Descriptive statistics for demographic characteristics by type of neighborhood

	total (n = 637)	low-SES/low-walk (n = 139)	low-SES/high-walk (n = 158)	high-SES/low-walk (n = 169)	high-SES/high-walk (n = 171)
**Age: mean (SD)**	14.6 (0.9)	14.5 (0.9)	14.6 (1.0)	14.5 (0.9)	14.6 (0.9)

**Gender: %**					

Male	49.6	50.4	50.6	47.3	50.3

Female	50.4	49.6	49.4	52.7	49.7%

**Educational level: %**					

**Mother:**					

Less than high school	9.9	19.8	16.7	2.0	3.9

Completed high school	25.1	39.7	17.4	28.9	17.5

Completed college	40.4	33.6	31.9	45.6	48.1

Completed University	24.6	6.9	34.1	23.5	30.5

**Father:**					

Less than high school	7.5	18.6	7.9	2.1	4.0

Completed high school	36.0	54.9	24.6	36.4	31.1

Completed college	26.6	19.5	35.4	30.8	29.1

Completed University	29.8	7.1	42.1	30.8	35.8

**Employment status: %**					

Both employed	68.5	61.6	56.5	78.8	75.5

One parent unemployed	26.7	29.7	36.4	20.6	20.9

Both unemployed	4.2	8.7	7.1	0.6	3.7

Descriptive statistics for the outcome variables are given in Table 2. The objectively measured accelerometer data revealed that the adolescents engaged in on average 33.0 (23.7) minutes/day of moderate to vigorous physical activity. The objectively determined average activity level was 401.8 (148.5) counts/minute. The adolescents reported on average 9.7 (11.7) minutes/day of walking during leisure time, 8.2 (10.7) minutes/day of cycling during leisure time, 11.5 (14.5) minutes/day of walking and cycling to and from school and 21.8 (24.1) minutes/day of sports during leisure time.

**Table 2:** Descriptive statistics for the outcome variables for the total group and by type of neighborhood

	total	low-SES/low-walk	low-SES/high-walk	high-SES/low-walk	high-SES/high-walk
**Accelerometer: mean min/day (SD)**	**(n = 513)**	**(n = 100)**	**(n = 137)**	**(n = 129)**	**(n = 147)**

MVPA	33.0 (23.7)	27.2 (19.9)	34.7 (23.9)	33.9 (22.3)	34.5 (26.4)

Counts/min	401.8 (148.5)	382.3 (161.2)	422.2 (164.0)	395.7 (140.8)	401.3 (128.6)

**FPAQ: mean min/day (SD)**	**(n = 615)**	**(n = 134)**	**(n = 150)**	**(n = 163)**	**(n = 168)**

Walking during leisure time	9.7 (11.7)	13.2 (14.0)	13.7 (13.7)	7.0 (8.8)	6.0 (7.5)

Cycling during leisure time	8.2 (10.7)	8.4 (11.1)	10.5 (12.7)	8.1 (10.2)	6.2 (8.4)

Active transport to and from school	11.5 (14.5)	11.0 (15.4)	13.2 (11.9)	12.1 (18.5)	9.7 (11.1)

Sports participation	21.8 (24.1)	17.9 (23.2)	23.1 (27.8)	22.1 (22.5)	23.6 (22.3)

### Associations of physical activity with neighborhood walkability and neighborhood SES

#### Objectively measured physical activity

**Figure 2:**
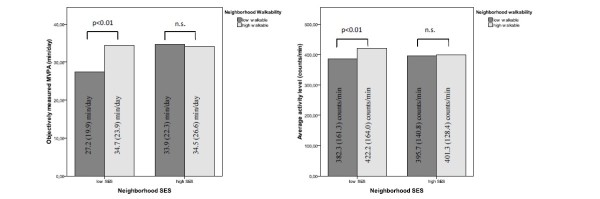
**Main effects of neighborhood walkability for objectively PA for low- and high-SES neighborhoods**. Results of the multivariate multi-level regression analyses testing the main effects of neighborhood walkability for objectively measured moderate to vigorous PA (MVPA) and average activity level for low- and high-SES neighborhoods separately.

Among adolescents living in low-SES neighborhoods, we found an association between neighborhood walkability and objectively measured moderate to vigorous physical activity (p < 0.01), whereas among adolescents living in high-SES neighborhoods we found no association. In low-SES neighborhoods, adolescents living in high-walkable neighborhoods performed more moderate to vigorous physical activity than adolescents living in low-walkable neighborhoods (34.7 (23.9) minutes/day and 27.2 (19.9) minutes/day respectively). (Table 3 and Figure 2)

**Table 3:** Multivariate multi-level regression analyses of the association between neighborhood walkability, neighborhood SES and physical activity

	Objectively measured		Self-reported			
	**MVPA**	**CPM**	**Active transport to and from school**	**Walking during leisure time**	**Cycling during leisure time**	**Sport during leisure time**

**MODEL 1**						

Educational attainment mother						

*less than high school (ref.)*						

completed high school	0.124(0.076)	0.036(0.034)	-0.120(0.125)	-0.206(0.102)	0.063(0.109)	0.234(0.133)

completed college	0.085(0.079)	0.012(0.035)	-0.027(0.130)	-0.169(0.107)	0.174(0.113)	0.292(0.138)

completed university	0.059(0.086)	0.001(0.038)	-0.060(0.143)	-0.198(0.117)	0.096(0.124)	0.370(0.152)

Educational attainment father						

*less than high school (ref.)*						

completed high school	-0.156(0.084)	-0.053(0.037)	-0.048(0.129)	-0.030(0.106)	-0.180(0.114)	-0.106(0.141)

completed college	-0.194(0.090)	-0.053(0.040)	0.178(0.142)	-0.186(0.116)	-0.118(0.124)	-0.108(0.154)

completed university	-0.184(0.094)	-0.075(0.042)	0.015(0.148)	-0.163(0.122)	-0.160(0.130)	-0.145(0.160)

Parental employment						

*both parents unemployed (ref.)*						

one of the parents employed	0.078(0.100)	0.044(0.044)	0.313(0.154)	-0.122(0.131)	0.169(0.134)	-0.162(0.166)

Both parents employed	0.206(0.098)	0.083(0.044)	0.458(0.152)	-0.151(0.129)	0.206(0.132)	0.048(0.163)

**WALK**						

***low-walk (ref.)***						

**high-walk**	0.091(0.035)*	0.053(0.017)**	**0.151(0.81)**	**0.131(0.099)**	**-0.014(0.067)**	**0.096(0.073)**

**MODEL 2**						

Educational attainment mother						

*less than high school (ref.)*						

completed high school	0.090(0.077)	0.024(0.034)	-0.123(0.125)	-0.211(0.102)	0.074(0.109)	0.201(0.133)

completed college	0.064(0.080)	0.007(0.036)	-0.009(0.131)	-0.160(0.107)	0.187(0.113)	0.263(0.139)

completed university	0.044(0.087)	-0.000(0.039)	-0.036(0.143)	-0.185(0.118)	0.107(0.124)	0.350(0.153)

Educational attainment father						

*less than high school (ref.)*						

completed high school	-0.155(0.085)	-0.051(0.038)	-0.035(0.129)	-0.025(0.106)	-0.176(0.114)	-0.105(0.141)

completed college	-0.192(0.091)	-0.050(0.041)	0.205(0.142)	-0.175(0.117)	-0.111(0.124)	-0.105(0.153)

completed university	-0.173(0.094)	-0.067(0.042)	0.049(0.148)	-0.146(0.122)	-0.154(0.129)	-0.134(0.159)

Parental employment						

*both parents unemployed (ref.)*						

one of the parents employed	0.057(0.101)	0.037(0.045)	0.317(0.154)	-0.117(0.130)	0.179(0.134)	-0.178(0.166)

Both parents employed	0.179(0.099)	0.073(0.044)	0.464(0.152)	-0.146(0.129)	0.220(0.132)	0.022(0.163)

**SES**						

***low-SES (ref.)***						

**high-SES**	**0.059(0.037)**	**0.013(0.018)**	**-0.154(0.081)**	-0.189(0.093)*	**-0.083(0.065)**	**0.102(0.073)**

**MODEL 2**						

Educational attainment mother						

*less than high school (ref.)*						

completed high school	0.121(0.075)	0.035(0.034)	-0.101(0.125)	-0.193(0.102)	0.079(0.109)	0.217(0.134)

completed college	0.081(0.078)	0.011(0.035)	-0.001(0.130)	-0.150(0.107)	0.194(0.113)	0.267(0.139)

completed university	0.050(0.085)	-0.001(0.035)	-0.037(0.143)	-0.180(0.117)	0.113(0.124)	0.347(0.152)

Educational attainment father						

*less than high school (ref.)*						

completed high school	-0.185(0.083)	-0.061(0.037)	-0.047(0.129)	-0.034(0.106)	-0.184(0.114)	-0.111(0.141)

completed college	-0.238(0.090)	-0.065(0.040)	0.185(0.143)	-0.192(0.117)	-0.125(0.125)	-0.118(0.155)

completed university	-0.237(0.093)	-0.089(0.042)	0.019(0.149)	-0.172(0.122)	-0.171(0.131)	-0.153(0.162)

Parental employment						

*both parents unemployed (ref.)*						

one of the parents employed	0.062(0.099)	0.040(0.044)	0.319(0.153)	-0.121(0.130)	0.171(0.134)	-0.171(0.166)

Both parents employed	0.187(0.097)	0.077(0.044)	0.474(0.151)	-0.145(0.129)	0.217(0.132)	0.032(0.163)

WALK						

*low-walk (ref.)*						

high-walk	0.230(0.053)	0.098(0.026)	0.193(0.113)	0.245(0.129)	0.075(0.092)	0.097(0.107)

SES						

*low-SES (ref.)*						

high-SES	0.187(0.052)	0.057(0.025)	-0.103(0.108)	-0.052(0.132)	0.003(0.090)	0.102(0.103)

**WALK × SES**	-0.228(0.068)***	-0.073(0.034)*	**-0.089(0.149)**	**-0.262(0.182)**	**-0.170(0.123)**	**0.005(0.140)**

Model 1,2 and 3 were corrected for educational attainment mother and father and parental employment

*p < 0.05, ** p < 0.01, *** p < 0.001

^1^ WALK: neighborhood walkability (low/high neighborhood walkability)

^2 ^SES: neighborhood socio-economic status (low/high neighborhood SES)

^3 ^MVPA: moderate to vigorous physical activity

^4 ^CPM: counts/minute

Similar to accelerometer-based moderate to vigorous physical activity, we found an association between neighborhood walkability and the objectively determined average activity level expressed in counts/minute among adolescents living in low-SES neighborhoods (p < 0.01), whereas among adolescents living in high-SES neighborhoods we found no association. In low-SES neighborhoods, adolescents living in high-walkable neighborhoods achieved a higher average activity level than adolescents living in low-walkable neighborhoods (422.2 (164.0) counts/minute and 382.3 (161.3) counts/minute respectively). (Table 3 and Figure 2)

Analyses of the moderating effect of gender showed that gender did not moderate any association between neighborhood walkability, neighborhood SES and moderate to vigorous physical activity or the average activity level.

#### Self-reported physical activity

For self-reported minutes/day of walking for transport during leisure time we found an association with neighborhood SES (p < 0.05). Adolescents living in low-SES neighborhoods reported on average more minutes/day walking for transport during leisure time than adolescents living in high-SES neighborhoods (13.4 min/day (13.8) vs. 6.5 min/day (8.2) respectively). No association between neighborhood walkability and self-reported minutes/day of walking for transport during leisure time was found.

For the reported minutes/day cycling during leisure time, active transport to and from school and sports during leisure time, no association was found with neighborhood walkability nor with neighborhood SES.

Gender did not moderate any association between neighborhood walkability, neighborhood SES and walking for transport during leisure time, cycling during leisure time, active transport to and from school and sports during leisure time.

## Discussion

The main finding of the present study was that for 13-15 year old Belgian adolescent boys and girls the average activity level and the mean minutes moderate to vigorous physical activity per day were associated with neighborhood walkability, but this association was moderated by neighborhood SES. Only in low-SES neighborhoods, adolescent boys and girls living in high-walkable neighborhoods performed more moderate to vigorous physical activity (+ 7.4 min/day) and achieved a higher average activity level (+ 39.9 counts/min) than adolescent boys and girls living in low-walkable neighborhoods. Among adolescents living in high-SES neighborhoods, no association was found between neighborhood walkability and accelerometer-based physical activity. For the self-reported variables-active transport to and from school, walking and cycling for transport during leisure time and sport during leisure time-no association with neighborhood walkability was found. These results are an indication that the main conclusion of the PLACE, NQLS, BEPAS and SNAP studies, that adults living in high-walkable neighborhoods reach higher levels of physical activity, cannot be extended to the overall group of 13-15 year old Belgian adolescents.

The contrasting results of the BEPAS-Y and the BEPAS adult [32] studies, conducted in the same neighbourhoods, are in particular noteworthy. The BEPAS adult study documents large differences in accelerometer-based moderate to vigorous physical activity across walkability groups, in both low- and high-SES neighborhoods. Given the similarity of environments and measures, questions can be raised about the different responses of adolescents and adults to their built environment.

Neighbourhood "walkability" refers to the ability to walk or cycle to nearby destinations. The key elements of "walkability" are determined based on adult research investigating the specific neighborhood environmental attributes that are associated with higher levels of walking and cycling for transport [38,39]. Walkability has been consistently found to be associated with walking for transport on three continents so far [30-33,54], as well as with cycling for transport [32]. In comparison with adult physical activity behavior, adolescent physical activity behavior is characterized by a larger variation in types of physical activity. Active transport forms only a fraction of adolescents' total physical activity behavior. Given this, the possibility exists that the construct "walkability" is not as relevant for adolescents. In the current evidence-base, the specific built environmental attributes that characterize a walkable neighborhood, are less consistently associated with adolescents' physical activity [29]. In some adolescent studies connectivity even showed an inverse association with physical activity [29]. Including connectivity in the walkability-index for adolescents could therefore negate some of the effects.

Considering the multidimensional character of neighborhood environments, it could be that among youth, other built environment attributes are the key elements of neighborhoods conducive to an active lifestyle. It could also be that family rules could interact with built environment and social environment features in influencing adolescent physical activity. Thus, further studies are needed to determine the specific environmental attributes characterizing the ability for youth to be active in a neighborhood. Parallel to research in adults, those attributes can be used to create an index that refers to the ability of youth to be active in their neighborhood, e.g. an "activability" index.

In the development of an "activability" index for youth it may be advisable to take into account the diverse physical activity domains adolescents are participating in. As stated by Giles-Corti et al. [55] environmental attributes are expected to have effects that vary by physical activity domain. In particular, walkability is expected to be related to active transportation, and a recent review confirms that proximity to parks and recreation facilities was one of the most consistent correlates of adolescent physical activity [29]. The present study did not include measures of recreation environments expected to be related to leisure time physical activity. Thus, an "activability" index likely needs to include recreation environment attributes as well. Due to large variations in built environments across countries and continents, an "activability" index may need to be tailored to each context. Research to identify built environmental attributes that have beneficial effects on physical activity in adults and youth is of utmost importance to inform policy makers and urban planners in making well-considered decisions concerning built environmental redevelopments of existing neighborhoods and planning of new neighborhoods.

Surprisingly, previous studies that found an interaction between neighborhood walkability and neighborhood SES showed a stronger association between neighborhood walkability and physical activity in high-income neighborhoods [31,37]. Part of the explanation for the interaction found in the present study may be attributed to economic factors. As stated by Stalsberg et al. [56] the observed SES moderating effect could be explained by varying ability to deal with the financial outlay that certain activities require (e.g. sport material, membership fees). Most adolescents living in high-SES neighborhoods have the ability to participate in activities that require financial outlay, in contrast to adolescents living in low-SES neighborhoods. Consequently, adolescents from high-SES neighborhoods are less dependent on their neighborhood environment to be active than their peer group from low-SES neighborhoods. This explanation is supported by the objectively measured levels of physical activity. The average activity level and mean number of minutes moderate to vigorous physical activity were lowest in the low-SES/low-walkable neighborhoods. Since there is evidence that low-SES adolescents often have lower levels of physical activity [57,58] and that SES shows a clear inverse relationship with levels of overweight and obesity [59,60], the present study's finding involve several implications for public policy and the results may be important for future environmental interventions or governmental initiatives. Improving income and education in low-SES communities is an ultimate solution. However, interim improvements can be based on emerging findings in the US that low-SES neighborhoods have numerous environmental deficits that are not reflected in walkability [61,62]. Low-income neighborhoods, regardless of walkability, were disadvantaged in access to recreation facilities, walking/cycling facilities, aesthetics, pedestrian/traffic safety, and crime safety [62]. Thus, if such environmental disparities are documented in Belgium, interventions could be targeted at low-income neighborhoods to provide more recreation facilities, improve walking/cycling facilities, enhance aesthetics by planting trees and removing graffiti, increase safety of street crossing, and institute more effective crime control methods. Walking for transport during leisure time was the only outcome for which an association with neighbourhood SES was found. Adolescent boys and girls living in low-SES neighbourhoods reported twice as much walking during leisure time than adolescent boys and girls living in high-SES neighbourhoods (13.4 (13.8) min/day vs. 6.5 (8.2) min/day). As stated by Ross et al. (2008), neighborhoods characterized by a higher level of poverty may have a culture in which people are outside on the streets, walk to visit someone, talk out on the street or just hang out on the street [53]. This culture may encourage adolescents to walk for transport during leisure time. Another possible explanation is that lower-SES households have less access to automobiles. The lack of association between neighborhood SES and the other physical activity variables may be explained by a finding by Voorhees et. al. [36]. In that study no association was found between SES and accelerometer-based physical activity in adolescent girls. However, some qualitative differences in types and location of activities between low- and high-SES girls were found. Lower-SES girls achieved higher levels of moderate to vigorous physical activity at home whereas higher-SES girls were more likely to participate in moderate to vigorous physical activity at school or community facilities. Furthermore, low-SES girls reported less moderate to vigorous physical activity in organized activity and were more involved in informal and spontaneous activities in comparison with high-SES girls. The Voorhees et al. (2009) results indicate that low-SES girls do more of their physical activity near home, suggesting that neighborhood environments could be more important for low-SES adolescents.

### Limitations and strengths

Limitations of the present study included the cross-sectional study design which does not permit causal inferences. Second, despite stratified recruitment in higher- and lower-SES neighborhoods, education levels of parents were generally high. This likely reflects both high education levels of the university city and some recruitment bias. Third, neighborhoods were defined using existing statistical sectors. In that way artificial neighborhoods were created. A possible consequence of this method is that those artificial neighborhoods are not compatible with how respondents would define their own neighborhood. Neighborhood-level indicators of walkability and SES are relatively crude indicators, and individual buffers may more accurately reflect the environments that adolescents are exposed to. Strengths of the present study included the use of both objective and self-reported measures of physical activity. The average activity level, expressed in accelerometer counts/minute, gives an indication of the total level of physical activity. This outcome is based on the raw data provided by the accelerometer and is not dependent of processing differences. The FPAQ was used to collect information about adolescents' physical activity behavior in diverse domains. Questionnaire answers may be biased by social desirability, but to reduce over reporting, interviewers were trained to question high reports. Second, GIS databases were used to identify neighborhoods that maximized variation in walkability, and Belgian census data were used to identify neighborhoods of low and high-SES. Third, this study included a large sample of adolescent boys and girls. Finally, the study design and protocol was similar to PLACE, BEPAS, NQLS and SNAP studies in adults and importantly was conducted in the same neighborhoods as the BEPAS Adult study.

## Conclusions

The main conclusion of the BEPAS-Youth was that overall, in adolescent boys and girls aged 13-15 years, the association between neighborhood walkability and physical activity depended on neighborhood SES. There was an association between neighborhood walkability and objectively measured physical activity only among girls and boys living in low-SES neighborhoods. The discrepancies between the results of this study of adolescents, a similar study of Belgian adults, and the results of previous international studies in adults and adolescents highlight the need to strengthen the evidence among adolescents. Generally, built environment attributes are less consistently related to physical activity of adolescents than adults. Future investigations should determine the key environmental attributes of an active living neighborhood with a particular relevance for youth. Based on those key attributes, an important next step is to create an "activability" index for youth, which could be used for research and to identify neighborhoods that are less supportive of physical activity and in need of interventions.

## Competing interests

The authors declare that they have no competing interests.

## Authors' contributions

All authors have made substantial contributions to the design of the study. FDM collected the data, conducted the statistical analysis and drafted the manuscript. DVD, GC, IDB and BD helped to prepare the data collection, provided feedback during the process, participated in the interpretation of the data and provided critical comments on the manuscript. JFS revised the draft for important intellectual content. All authors read and approved the final version of the manuscript.

## Pre-publication history

The pre-publication history for this paper can be accessed here:

http://www.biomedcentral.com/1471-2458/12/7/prepub

## References

[B1] World Health Organization Global Stragey on diet, Physical activity and Health 2004 Geneva: Geneva, Zwitzerland: World Health Organization

[B2] Strong WB Malina RM Blimkie CJ Daniels SR Dishman RK Gutin B Hergenroeder AC Must A Nixon PA Pivarnik JM Evidence based physical activity for school-age youth J Pediatr 2005 146 732 737 10.1016/j.jpeds.2005.01.055 15973308

[B3] U.S. Department of Health and Human Services http://www.health.gov/paguidelines/guidelines/default.aspx 10.3109/15360288.2015.1037530 26095483

[B4] Janssen I Leblanc AG Systematic review of the health benefits of physical activity and fitness in school-aged children and youth Int J Behav Nutr Phys Act 2010 7 40 10.1186/1479-5868-7-40 20459784 PMC2885312

[B5] Riddoch CJ Bo AL Wedderkopp N Harro M Klasson-Heggebo L Sardinha LB Cooper AR Ekelund U Physical activity levels and patterns of 9- and 15-yr-old European children Med Sci Sports Exerc 2004 36 86 92 10.1249/01.MSS.0000106174.43932.92 14707773

[B6] Currie C Gabhainn SN Godeau E Roberts C Smith R Currie D Picket W Richter M Morgan A Barnekow V Inequalities in young people's health. HBSC international report from the 2005/2006 survey 2008

[B7] Cavill N Kahlmeier S Racioppi F Physical activity and health in Europe: evidence for action 2006 World Health Organisation

[B8] Sisson SB Katzmarzyk PT International prevalence of physical activity in youth and adults Obes Rev 2008 9 606 614 10.1111/j.1467-789X.2008.00506.x 18647243

[B9] Biddle SJ Gorely T Stensel DJ Health-enhancing physical activity and sedentary behaviour in children and adolescents J Sports Sci 2004 22 679 701 10.1080/02640410410001712412 15370482

[B10] Kelder SH Perry CL Klepp KI Lytle LL Longitudinal tracking of adolescent smoking, physical activity, and food choice behaviors Am J Public Health 1994 84 1121 1126 10.2105/AJPH.84.7.1121 8017536 PMC1614729

[B11] van Mechelen W Twisk JW Post GB Snel J Kemper HC Physical activity of young people: the Amsterdam Longitudinal Growth and Health Study Med Sci Sports Exerc 2000 32 1610 1616 10994913 10.1097/00005768-200009000-00014

[B12] Sallis JF Owen N Fisher EB Glanz K, Rimer BB, Viswanath K Ecological Models of Health Behaviour Health Behavioour and Health Education: Theory, Research and Practice 2008 4 San Francisco: Jossey-Bass 462 484

[B13] Hsu YW Chou CP Nguyen-Rodriguez ST McClain AD Belcher BR Spruijt-Metz D Influences of social support, perceived barriers, and negative meanings of physical activity on physical activity in middle school students J Phys Act Health 2011 8 210 219 21415448 10.1123/jpah.8.2.210 PMC8098645

[B14] Sallis JF Prochaska JJ Taylor WC A review of correlates of physical activity of children and adolescents Med Sci Sports Exerc 2000 32 963 975 10795788 10.1097/00005768-200005000-00014

[B15] Pate RR Trost SG Felton GM Ward DS Dowda M Saunders R Correlates of physical activity behavior in rural youth Res Q Exercise Sport 1997 68 241 248 10.1080/02701367.1997.10608003 9294878

[B16] Voorhees CC Murray D Welk G Birnbaum A Ribisl KM Johnson CC Pfeiffer KA Saksvig B Jobe JB The role of peer social network factors and physical activity in adolescent girls Am J Health Behav 2005 29 183 190 15698985 10.5993/ajhb.29.2.9 PMC2507875

[B17] Lagarde F LeBlanc C Policy options to support physical activity in schools C J Public Health 2010 101 Suppl 2 S9 S13 10.1007/BF03405618 PMC6974204 21137137

[B18] Panter JR Jones AP van Sluijs EM Environmental determinants of active travel in youth: A review and framework for future research Int J Behav Nutr Phys Act 2008 5 34 10.1186/1479-5868-5-34 18573196 PMC2483993

[B19] Prins RG Oenema A Van Der Horst K Brug J Objective and perceived availability of physical activity opportunities: differences in associations with physical activity behavior among urban adolescents Int J Behav Nutr Phys Act 2009 6 70 10.1186/1479-5868-6-70 19832969 PMC2770555

[B20] Cohen DA Ashwood JS Scott MM Overton A Evenson KR Staten LK Porter D McKenzie TL Catellier D Public parks and physical activity among adolescent girls Pediatrics 2006 118 e1381 e1389 10.1542/peds.2006-1226 17079539 PMC2239262

[B21] Carver A Timperio AF Crawford DA Neighborhood road environments and physical activity among youth: the CLAN study J Urban Health 2008 85 532 544 10.1007/s11524-008-9284-9 18437579 PMC2443253

[B22] Saelens BE Sallis JF Black JB Chen D Neighborhood-based differences in physical activity: an environment scale evaluation Am J Public Health 2003 93 1552 1558 10.2105/AJPH.93.9.1552 12948979 PMC1448009

[B23] Lake A Townshend TG Alvanides S Obesogenic Environments 2010 Wiley-Blackwell

[B24] Leslie E Walkability of local communities: Using geographic information systems to objectively assess relevant environmental attributes Health Place 2006 13 111 122 16387522 10.1016/j.healthplace.2005.11.001

[B25] Kligerman M Sallis JF Ryan S Frank LD Nader PR Association of neighborhood design and recreation environment variables with physical activity and body mass index in adolescents Am J Health Promot 2007 21 274 277 10.4278/0890-1171-21.4.274 17375494

[B26] Patnode CD Lytle LA Erickson DJ Sirard JR Barr-Anderson D Story M The relative influence of demographic, individual, social, and environmental factors on physical activity among boys and girls Int J Behav Nutr Phys Act 2010 7 79 10.1186/1479-5868-7-79 21047429 PMC2991277

[B27] Van Dyck D Cardon G Deforche B De Bourdeaudhuij I Lower neighbourhood walkability and longer distance to school are related to physical activity in Belgian adolescents Prev Med 2009 48 516 518 10.1016/j.ypmed.2009.03.005 19285102

[B28] Maddison R Vander Hoorn S Jiang Y Mhurchu CN Exeter D Dorey E Bullen C Utter J Schaaf D Turley M The environment and physical activity: The influence of psychosocial, perceived and built environmental factors Int J Behav Nutr Phys Act 2009 6 19 10.1186/1479-5868-6-19 19331652 PMC2683167

[B29] Ding D Sallis JF Kerr J Lee S Rosenberg DE Neighborhood Environment and Physical Activity among Youth: A Review Am J Prev Med in press 10.1016/j.amepre.2011.06.036 21961474

[B30] Sallis JF Saelens BE Frank LD Conway TL Slymen DJ Cain KL Chapman JE Kerr J Neighborhood built environment and income: examining multiple health outcomes Soc Sci Med 2009 68 1285 1293 10.1016/j.socscimed.2009.01.017 19232809 PMC3500640

[B31] Owen N Cerin E Leslie E du Toit L Coffee N Frank LD Bauman AE Hugo G Saelens BE Sallis JF Neighborhood walkability and the walking behavior of Australian adults Am J Prev Med 2007 33 387 395 10.1016/j.amepre.2007.07.025 17950404

[B32] Van Dyck D Cardon G Deforche B Sallis JF Owen N De Bourdeaudhuij I Neighborhood SES and walkability are related to physical activity behavior in Belgian adults Prev Med 2010 50 S74 S79 19751757 10.1016/j.ypmed.2009.07.027

[B33] Sundquist K Eriksson U Kawakami N Skog L Ohlsson H Arvidsson D Neighborhood walkability, physical activity, and walking behavior: The Swedish Neighborhood and Physical Activity (SNAP) study Soc Sci Med 2011 10.1016/j.socscimed.2011.03.004 21470735

[B34] Boone-Heinonen J Evenson K Song Y Gordon-Larsen P Built and socioeconomic environments: patterning and associations with physical activity in U.S. adolescents Int J Behav Nutr Phys Act 2010 7 45 10.1186/1479-5868-7-45 20487564 PMC3152773

[B35] McCormack GR Giles-Corti B Timperio A Wood G Villanueva K A cross-sectional study of the individual, social, and built environmental correlates of pedometer-determined physical activity among elementary school children Int J Behav Nutr Phys Act 2011 8 30 10.1186/1479-5868-8-30 21486475 PMC3083320

[B36] Voorhees CC Catellier DJ Ashwood JS Cohen DA Rung A Lytle L Conway TL Dowda M Neighborhood socioeconomic status and non school physical activity and body mass index in adolescent girls J Phys Act Health 2009 6 731 740 20101916 10.1123/jpah.6.6.731 PMC2854409

[B37] Kerr J Rosenberg D Sallis JF Saelens BE Frank LD Conway TL Active commuting to school: Associations with environment and parental concerns Med Sci Sports Exerc 2006 38 787 794 10.1249/01.mss.0000210208.63565.73 16679998

[B38] Heath GW Brownson RC Kruger J Miles R Powell KE Ramsey LT The Task Force on Community Preventive Services The effectiveness of urban design and land use and transport policies and practices to increase physical activity: a systematic review J Phys Act Health 2006 3 1 55 76 10.1123/jpah.3.s1.s55 28834525

[B39] Gebel K King L Bauman A Vita P Gill T Rigby A Capon A Creating healthy environments: A review of links between the physical environment, physical activity and obesity Sydney: NSW Health Department and NSW Centre for Overweight and Obesity 2005

[B40] Frank LD Sallis JF Saelens BE Leary L Cain K Conway TL Hess PM The development of a walkability index: application to the Neighborhood Quality of Life Study Br J Sports Med 2010 44 924 933 10.1136/bjsm.2009.058701 19406732

[B41] John D Tyo B Bassett DR Comparison of four ActiGraph accelerometers during walking and running Med Sci Sports Exerc 2010 42 368 374 19927022 10.1249/MSS.0b013e3181b3af49 PMC2809132

[B42] Lee KY Macfarlane D Cerin E Do Three Different Generations of the Actigraph Accelerometer Provide the Same Output? ACSM 2010: American College of Sports Medicine (ACSM) 2010 Annual Meeting

[B43] Ottevaere C Huybrechts I De MF De B Cuenca-Garcia M De HS The use of accelerometry in adolescents and its implementation with non-wear time activity diaries in free-living conditions J Sports Sci 2010 1 11 21104522 10.1080/02640414.2010.521169

[B44] De Meester F De Bourdeaudhuij I Deforche B Ottevaere C Cardon G Measuring physical activity using accelerometry in 13-15-year-old adolescents: the importance of including non-wear activities Public Health Nutr 2011 1 10 21835080 10.1017/S1368980011001868

[B45] Santech Inc. Meterplus 4.2 2010

[B46] Corder K van Sluijs EM Goodyer I Ridgway CL Steele RM Bamber D Dunn V Griffin SJ Ekelund U Physical activity awareness of british adolescents Arch Pediatr Adolesc Med 2011 165 603 609 10.1001/archpediatrics.2011.94 24187480 PMC3812705

[B47] Rowlands AV Pilgrim EL Eston RG Patterns of habitual activity across weekdays and weekend days in 9-11-year-old children Prev Med 2008 46 317 324 10.1016/j.ypmed.2007.11.004 18162187

[B48] Puyau M Adolph A Vohra F Validation and calibration of physical activity monitors in children Obes Res 2002 10 150 157 10.1038/oby.2002.24 11886937

[B49] Corder K Ekelund U Steele RM Wareham NJ Brage S Assessment of physical activity in youth J Appl Physiol 2008 105 977 987 10.1152/japplphysiol.00094.2008 18635884

[B50] Trost SG Pate RR Freedson PS Sallis JF Taylor WC Using objective physical activity measures with youth: how many days of monitoring are needed? Med Sci Sports Exerc 2000 32 426 431 10.1097/00005768-200002000-00025 10694127

[B51] Trost SG McIver KL Pate RR Conducting accelerometer-based activity assessments in field-based research Med Sci Sports Exerc 2005 37 S531 S543 10.1249/01.mss.0000185657.86065.98 16294116

[B52] Philippaerts RM Matton L Wijndaele K Balduck AL De BI Lefevre J Validity of a physical activity computer questionnaire in 12- to 18-year-old boys and girls Int J Sports Med 2006 27 131 136 10.1055/s-2005-837619 16475059

[B53] Ross CE Mirowsky J Neighborhood socioeconomic status and health: Context of composition? City Community 2008 7 163 169 10.1111/j.1540-6040.2008.00251.x

[B54] Saelens BE Handy SL Built environment correlates of walking: a review Med Sci Sports Exerc 2008 40 S550 S566 10.1249/MSS.0b013e31817c67a4 18562973 PMC2921187

[B55] Giles-Corti B Timperio A Bull F Pikora T Understanding physical activity environmental correlates: increased specificity for ecological models Exerc Sport Sci Rev 2005 33 175 181 10.1097/00003677-200510000-00005 16239834

[B56] Stalsberg R Pedersen AV Effects of socioeconomic status on the physical activity in adolescents: a systematic review of the evidence Scand J Med Sci Sports 2010 20 368 383 10.1111/j.1600-0838.2009.01047.x 20136763

[B57] Borraccino A Socioeconomic Effects on Meeting Physical Activity Guidelines: Comparisons among 32 Countries Med Sci Sports Exerc 2009 41 749 756 10.1249/MSS.0b013e3181917722 19276860 PMC2663903

[B58] Ferreira I Van Der HK Wendel-Vos W Kremers S Van Lenthe FJ Brug J Environmental correlates of physical activity in youth-a review and update Obes Rev 2007 8 129 154 10.1111/j.1467-789X.2006.00264.x 17300279

[B59] Morgen CS Mortensen LH Rasmussen M Andersen AM Sorensen TI Due P Parental socioeconomic position and development of overweight in adolescence: longitudinal study of Danish adolescents BMC Public Health 2010 10 520 10.1186/1471-2458-10-520 20799987 PMC2940915

[B60] Shrewsbury V Wardle J Socioeconomic status and adiposity in childhood: a systematic review of cross-sectional studies 1990-2005 Obesity (Silver Spring) 2008 16 275 284 10.1038/oby.2007.35 18239633

[B61] Lovasi GS Hutson MA Guerra M Neckerman KM Built Environments and Obesity in Disadvantaged Populations Epidemiol Rev 2009 31 7 20 10.1093/epirev/mxp005 19589839

[B62] Sallis JF Slymen DJ Conway TL Frank LD Saelens BE Cain K Chapman JE Income disparities in perceived neighborhood built and social environment attributes Health Place 2011 17 1274 1283 10.1016/j.healthplace.2011.02.006 21885324

